# GLIPR-2 Overexpression in HK-2 Cells Promotes Cell EMT and Migration through ERK1/2 Activation

**DOI:** 10.1371/journal.pone.0058574

**Published:** 2013-03-13

**Authors:** Shaoguang Huang, Fei Liu, Qin Niu, Yi Li, Chang Liu, Lele Zhang, Danni Ni, Xiaoyun Pu

**Affiliations:** 1 Department of Clinical Laboratory, Xin Qiao Hospital, Third Military Medical University, Chong Qing, PR China; 2 Institute of Pathology, Southwest Hospital, Third Military Medical University, Chong Qing, PR China; University of Birmingham, United Kingdom

## Abstract

The epithelial-to-mesenchymal transition (EMT) of tubular epithelial cells in the adult kidney is one of the key events in renal interstitial fibrosis. Glioma pathogenesis related-2 (GLIPR-2) has been shown to be up-regulated in proximal tubular cells (PTCs) in the fibrotic kidney. However, the biological function of GLIPR-2 remains unknown. In this study, we found that GLIPR-2 expression is elevated in the kidney tissue samples of patients with diabetic nephropathy (DN). Human proximal renal tubular epithelial cells (HK-2 cells) were transfected with pcDNA3.0-GLIPR-2 and selected with G418. To identify the biological function of GLIPR-2, an epithelial-to-mesenchymal transition (EMT) PCR array analysis was performed, and genes that had statistically significantly altered expression levels with more than a two-fold difference compared with the pcDNA3.0-transfected HK-2 cells were considered. Key elements of the EMT process, such as E-cadherin and vimentin, were transcriptionally activated in the pcDNA3.0-GLIPR-2-transfected sublines. In addition, α-SMA gene expression, which is a marker of myofibroblasts, increased in the pcDNA3.0-GLIPR-2-transfected HK-2 cells. The cell migration assay demonstrated that the transfection of HK-2 with GLIPR-2 promoted cell migration following an EMT. Additionally, consistent with the effects of increased EGFR expression levels, we found that the activation of extracellular signal-regulated kinases 1 and 2 (ERK1/2) was highly elevated in the pcDNA3.0-GLIPR-2-transfected group. Our study demonstrates that GLIPR-2 overexpression in HK-2 cells can potentiate EMT-like processes in this cell type through the ERK1/2 signaling pathway. GLIPR-2 may be responsible for the development of renal fibrosis by increasing the accumulation of interstitial fibroblasts.

## Introduction

Renal fibrosis is a serious complication of chronic renal diseases (CRD) that is characterized by the excessive deposition of extracellular matrix (ECM) in the glomerular mesangial and tubulointerstitial region and can lead to end-stage renal failure [1]–[3]. While the etiology of renal fibrosis is not fully understood, myofibroblasts are believed to contribute to the synthesis of the ECM components and renal failure [4], [5]. It has been reported that the number of myofibroblasts correlates with the degree of tubulointerstitial fibrosis and the progression of renal insufficiency in CRD. Myofibroblasts, a group of smooth muscle-like fibroblasts, not only abundantly synthesize ECM components but also express alpha-smooth muscle actin (α-SMA), which controls cell motility [6], [7]. It has been reported that at least one third of the myofibroblasts are derived from the epithelial-to-mesenchymal transition (EMT) of renal proximal tubular cells (PTCs) in the fibrotic kidney [8]. Thus, EMT may play a central role in renal fibrosis. The data from *in vitro* studies have shown that, during EMT, PTCs lose their apical-basal polarity and epithelial cell adhesion molecules (E-cadherin) and acquire a myofibroblast phenotype by expressing α-SMA. The EMT is also characterized by enhanced cell migration and invasion [9]. Emerging evidence demonstrates that a large proportion of renal tubular epithelial cells express α-SMA in the diseased kidney, indicating the transdifferentiation of epithelial cells into myofibroblasts [10].

Glioma pathogenesis related-2 (GLIPR-2), whose aliases include GAPR-1 and C9orf19, is a conserved mammalian protein belonging to the pathogenesis related-1 (PR-1) family and is mainly expressed in peripheral leukocytes and the lung in humans [11]–[13]. In contrast to plant PR-1 proteins and other mammalian SCP/CAP domain-containing proteins, GLIPR-2 is a non-secretory protein because it lacks a signal peptide [12], [14]. It has been reported that GLIPR-2 is abundantly increased in PTCs during kidney fibrogenesis [11]. However, the biological functions of GLIPR-2 remain unknown.

The purpose of this study is to identify the role and the underlying mechanisms of GLIPR-2 expression in EMT. We overexpressed GLIPR-2 in the HK-2 cell line, which is an immortalized human cell line used as an *in vitro* model for human proximal tubular cells, and found that GLIPR-2 overexpression in HK-2 cells promoted the expression of EMT markers and cell migration through the activation of extracellular signal-regulated kinases 1 and 2 (ERK1/2). Thus, GLIPR-2 may play a critical role in the progression of kidney fibrosis.

## Methods

### Ethics Statement

Approval for this study was obtained from the Ethical Committee of Xinqiao Hospital of Third Military Medical University, Chongqing, China. In addition, the kidney paraffin sections of DN were obtained from five patients who underwent renipuncture, and normal kidney tissue was obtained from two who patients underwent the resection of a renal carcinoma. All of the patients provided written informed consent.

### Cell Culture

HK-2 cells (Institute of Biochemistry and Cell Biology, Shanghai, China), a permanent and well-characterized human proximal tubular cell line immortalized by transduction with human papilloma virus (HPV) 16 E6/E7 genes [15], were kindly provided by Dr. Yunjian Huang (Department of Nephrology, Renal Disease Research Center, Xinqiao Hospital, Third Military Medical University, Chongqing, China). HK-2 cells at passages 10–15 were cultured in DMEM/low glucose (Sigma-Aldrich, St. Louis, MO, USA)supplemented with 20% GIBCO™ fetal calf serum (FCS) (Invitrogen, Camarillo, CA, USA), 4 mM L-glutamine, 100 U/ml penicillin, 100 µg/ml streptomycin and 20 mM HEPES buffer (Sigma-Aldrich, St. Louis, MO, USA). The cells were grown in a humidified incubator at 37°C in an atmosphere of 5% CO_2_ and 95% air. After being cultured in fresh medium for another 24 h, the cells were harvested and processed for Western blot analysis and RT-PCR. For the migration assay, the cells were grown in the absence of serum in 6-well plates for 24 h prior to the experiments.

### Plasmid Construction, Transfection, and Hybridization to the Arrays

The full length GLIPR-2 sequence was cloned from the cDNA of human peripheral blood mononuclear cells using Oligo (dT) 15 primers (Promega, Madison, WI, USA) including a pair of *KpnI* and *EcoRI* linkers. The forward primer was 5′-CGCGGTACCATGGGCAAGTCAGCATCCAAACAGT-3′ (*KpnI* site underlined), and the reverse primer was 5′-GGCGAATTCTTACTTCTTCGGCGGCAGGACGTT-3′ (*EcoRI* site underlined). The primers were used to amplify the GLIPR-2 coding sequence. Using the two linkers, the eukaryotic expression vector pcDNA3.0-GLIPR-2 was constructed and then transformed into DH5a *E. coli* cells (TAKARA Biotechnology Co. Ltd, Dalian, China). The orientation of the insert was verified by sequencing. The *E. coli* cells transformed with pcDNA3.0-GLIPR-2 or pcDNA3.0 were inoculated into an LB medium containing ampicillin (50 µg/ml). The plasmid was extracted by alkaline lysis, purified with PEG (Shanghai Biocolor BioScience & Technology Company, Shanghai,China), and transfected into HK-2 cells using the Lipofectamine™ 2000 transfection reagent (Invitrogen, Camarillo, CA, USA) according to the manufacturer’s instructions.

The HK-2 cells were plated at a density of 1×10^5^ cells per well into a 6-well plate with 2 ml DMEM/low glucose containing 20% FBS and incubated for 24 h until they reached 80–90% confluence. After rinsing the cells with serum-free medium without antibiotics, we added the pcDNA3.0-GLIPR-2-Lipofectamine mixture or the pcDNA3.0-Lipofectamine mixture as a control. After 48 h, to select for transfected cells, 500 µg/ml G418 (Biosharp, Korea) was administered for 2 weeks and until antibiotic-resistant colonies were observed. Untreated HK-2 cells were used as a control, and no viable cells were observed at the same G418 concentration. One drug-resistant colony from each of the two groups was isolated, propagated in medium containing 200 µg/ml G418 and used for subsequent experiments. Phenotypic changes in the HK-2 cells with GLIPR-2 overexpression were examined with the commercially available SABiosciences RT^2^ Profiler™ PCR Array of Human Epithelial to Mesenchymal Transition (PAHS-090A) (QIAGEN, Hilden, Germany), which was performed at Shanghai KangChen Bio-tech (Shanghai, China) as described in the manufacturer’s protocol.

The non-myristoylated GLIPR-2 plasmid was manufactured by a subdivision of Invitrogen (Shanghai, China). Briefly, the pair of mutation primers was: 5′- CTACCCAAGCTTGCCACCATGGCTAAGTCAGCTTCCAAACAG-3′ (HindIII site underlined) and 5′- CTACCGCTCGAGTTACTTCTTCGGCGGCAGGACGTTT-3′ (XhoI site underlined). The non-myristoylated GLIPR-2 sequence was cloned into pcDNA3.0, and the results were identified by sequencing. The transfection was carried out as previously described.

ERK1/2 siRNA and control siRNA were purchased from Cell Signaling Technology (Danvers, MA, USA ), and 48 to 72 hrs prior to cell lysis, 100 nM siRNA was transfected into the HK-2 cells that overexpressed GLIPR-2.

### Real-time Quantitative PCR (RT-qPCR)

Total RNA was extracted from the HK-2 cells with the TRIZOL™ reagent (Invitrogen, Camarillo, CA, USA). The total RNA (1 µg) from each sample was reverse-transcribed into cDNA with the M-MLV reverse transcriptase (Promega, Madison, WI, USA) according to the manufacturer’s instructions. Real-time qPCR was performed with SYBR Green (Toyobo, Osaka, Japan) on an ABI 7500 Real-Time PCR System (Applied Biosystems, Foster City, CA, USA) according to the manufacturer’s protocol. The primers were designed using Primer Premier 5.0 Software (PREMIER Biosoft International, Palo Alto, CA, USA) and are listed in Table 1. The amplification was conducted under the following conditions: 95°C for 30 s, 95°C for 5 s, 62°C for 10 s, and 72°C for 45 s for 40 cycles. Nuclease-free water (TIANGEN, Beijing, China) was substituted for the cDNA as a negative control in each PCR reaction. The relative gene expression was determined using the 2−ΔΔCt method according to the manufacturer’s recommended protocol.

**Table 1:** Gene Primer Sequences Used for RT-qPCR.

Gene		Primer sequences	Amplicon size (bp)	Gene accession no.
GLIPR-2	forward reverse	5′-ATCAAGAACTATAACTTCCAGCAGC-3′ 5′-AGCCCTCATTGACAACATTCC-3′	172	NM_022343
α-SMA	forward reverse	5′-CCCTTGAGAAGAGTTACGAGTTG-3′ 5′-ATGATGCTGTTGTAGGTGGTTTC-3′	145	NM_001613
EGFR	forward reverse	5′-TCGGTGTAAACGTTGCAAAA-3′ 5′-GACCACGGAGGATAGTATGAGC-3′	140	NM_005228
PDGFRB	forward reverse	5′-CTGCCCTGCCTGAAGCTC-3′ 5′-GTTTTCTTGCCTCCTAAGCC-3′	168	NM_005985
CDH1	forward reverse	5′-CTGACACACCCCCTGTTGGT-3′ 5′-GGTGAATTCGGGCTTGTTGT-3′	200	NM_004360
VIM	forward reverse	5′-TGCTTCTCTGGCACGTCTTG-3′ 5′-GGACATGCTGTTCCTGAATCTG-3′	128	NM_003380
VCAN	forward reverse	5′-GATCCCTAAAATGGCGAACATG-3′ 5′-CCAAAATGACTGAACGGTGGTC-3′	130	NM_004385
IL1RN	forward reverse	5′-GAGCTTCTGGCACTTGGAGACT-3′ 5′-TAGGGAACTTTGCACCCAACAT-3′	195	NM_000577
β-actin	forward reverse	5′-TGACGTGGACATCCGCAAAG-3′ 5′-CTGGAAGGTGGACAGCGAGG-3′	205	NM_001614

### Western Blotting

Cellular proteins were obtained by lysing the cells in ice-cold lysis buffer (100 mM Tris (pH 8), 2 mM EDTA, 100 mM NaCl, and 1% Triton X-100) containing complete EDTA-free protease inhibitors from Roche Diagnostics. Equal amounts of the proteins (50 µg) were denatured for 10 min at 95°C and loaded onto a 12% SDS-polyacrylamide gel for electrophoresis. After electrophoresis, the separated proteins were transferred to a PVDF membrane (Bio-Rad, Hercules, CA, USA) by electroblotting. The membrane was blocked with 5% non-fat milk in PBS and 0.1% Tween-20 for 2 hrs at room temperature. The membrane was then incubated in the presence of primary antibodies overnight at 4°C, followed by detection with an HRP-conjugated secondary antibody (ZSGB Biotechnology, Beijing, China) (1∶2000) and an enhanced electrochemiluminescence (ECL) detecting reagent (GE Healthcare, Piscataway, NJ, USA). Mouse monoclonal antibodies against E-cadherin, vimentin, α-SMA and GLIPR-2 were obtained from Santa Cruz Biotechnology, Inc. (Santa Cruz, CA, USA) and used at a 1∶500 dilution, and anti p44/42 or phospho-p44/42 MAPK (ERK1/2) monoclonal antibodies were obtained from Cell Signaling Technology (Danvers, MA, USA) and used at a 1∶2000 dilution.

### Migration Assay

Cell migration was examined using 24-well culture plates with 8-µm pore size Millicell cell culture inserts (Millipore, Volketswil, Switzerland). Briefly, we seeded 1×10^5^ HK-2 cells that had been deprived of serum for 24 hrs onto the upper compartment of the Millicell prior to adding stimuli to the lower chamber under serum-free conditions. After an 8-hr incubation, the upper surfaces of the filters were gently wiped with cotton swabs. The filters were then washed with PBS, fixed with methanol, and stained with 0.1% crystal violet. The number of migrating cells was counted under a light microscope in six random high-power (200×) fields (n = 6) per filter. Three separate experiments were performed for each assay.

### Immunofluorescence and Immunohistochemistry

Briefly, the cells were grown on coverslips in 24-well plates, serum-deprived for 12 hrs, and then washed with PBS, fixed with 4% paraformaldehyde and permeabilized in 0.1% Triton X-100/PBS 1X for 10 min. The cells were incubated with the anti-GLIPR-2 primary antibody (Santa Cruz Biotechnology, CA, USA) (1∶200) at 4°C overnight and then labeled with the FITC-conjugated secondary antibody (ZSGB Biotechnology, Beijing, China) (1∶2000) at room temperature for 30 min. The cells were washed before nuclear staining with 1 µg/mL Hoechst 33342 (Calbiochem, Bad Soden, Germany). Images were obtained with a Leica TCS-SPE confocal microscope (Leica, Mannheim, Germany).

### Statistical Analysis

Statistical analysis was performed using a one-way analysis of variance (ANOVA), and all of the values were expressed as the means ± SD. The statistical package SPSS11.0 (SPSS Inc., Chicago, USA) was used for all analyses. *P*<0.05 was considered statistically significant.

## Results

### GLIPR-2 is Overexpressed in the Fibrotic Kidney Accompanied by ERK Activation

**Figure 1:**
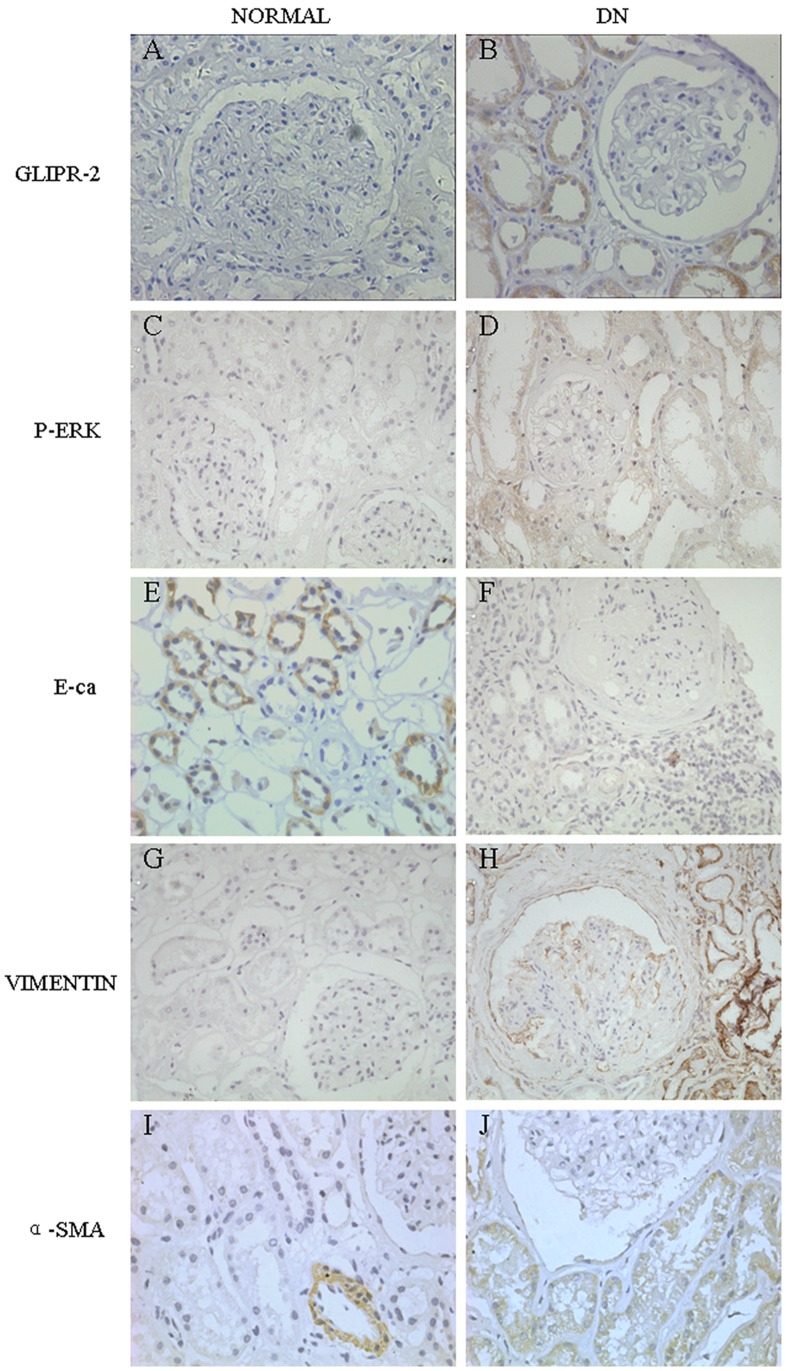
GLIPR-2 expression increases in PTCs specifically in the DN kidney. Sections of kidney from DN patients were stained with anti-GLIPR-2 (GLIPR-2), anti-p-ERK1/2 (P-ERK), anti-E-cadherin (E-ca), anti-vimentin (VIMENTIN) and anti-α-smooth muscle actin (α-SMA) antibodies. The GLIPR-2 staining was increased in PTCs of DN kidney (A) compared with normal kidney (B). In addition, p-ERK1/2 was also increased in the PTCs of DN kidney (C and D). The EMT marker E-cadherin decreased but the EMT markers vimentin and α-SMA increased in the DN kidney compared with control (E to J).

To identify a GLIPR-2 increase in the PTCs of the fibrotic kidney, we detected the expression of GLIPR-2 in kidney tissue paraffin sections from patients with DN. As shown in Figure 1, the expression of GLIPR-2 was found to be significantly increased in the PTCs of DN patients compared with normal tissue (Figure 1A and 1B). Furthermore, EMT biomarkers, such as E-cadherin, vimentin and α-SMA, were also altered in the PTCs of DN patients: the protein level of the epithelium marker E-cadherin decreased, while the mesenchymal marker vimentin displayed an increased protein level (Figure 1E to 1J). The data suggest that GLIPR-2 expression may be correlated with an EMT. Additionally, the result from the microarray analysis indicated that EGFR increased and PDGFRB decreased, suggesting that the extracellular signal-regulated kinase 1/2 (ERK1/2) signaling pathway downstream of the EGFR may be involved in the EMT process. Therefore, we analyzed the level of p-ERK in the paraffin sections from the DN patients and found that it was higher than the p-ERK level in the normal kidney tissue controls (Figure 1C and 1D).

### Establishment of HK-2 Cells with Stably Overexpressed GLIPR-2

**Figure 2:**
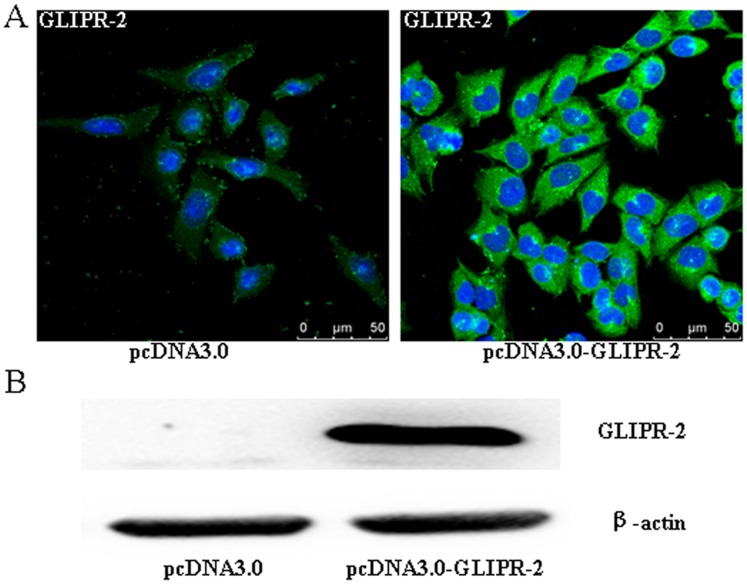
GLIPR-2 expression in pcDNA3.0-GLIPR-2-transfected HK-2 cells. (A) Immunofluorescence shows a high level of GLIPR-2 expression in the pcDNA3.0-GLIPR-2-transfected HK-2 cells, which is mainly cytoplasmic. (B) Western blot analysis of the GLIPR-2 expression (18 kDa).

To determine the effects of GLIPR-2 on PTCs, HK-2 cells were transfected with pcDNA3.0 or pcDNA3.0-GLIPR-2, and the sub-clonal cells were established by G418 selection. The levels of GLIPR-2 mRNA and protein expression were examined by reverse transcriptase RT-PCR, immunofluorescence and Western blotting. As shown in Figure 2, GLIPR-2 could not be detected in the parental cells stably transfected with the empty vector (mock-transfected cells), whereas GLIPR-2 expression significantly increased in the cells stably transfected with the pcDNA3.0-GLIPR-2 plasmid; the expression of GLIPR-2 mRNA is shown in Figure S1. These results indicate that the recombinant plasmid used in this study is efficient in expressing GLIPR-2 in the HK-2 cells.

### EMT-related Genes are Altered in GLIPR-2-overexpressing HK-2 Cells

To investigate whether GLIPR-2 affects the expression of genes that regulate an EMT, we performed a genetic analysis of the GLIPR-2-overexpressing HK-2 cells and control cells with the SABiosciences RT^2^ Profiler™ PCR Array of Human EMT. Among the 84 genes encoding the mediators and/or downstream effectors of EMT, we found 8 up-regulated genes (CTNNB1, EGFR, ITGAV, SNAI2, SPARC, STEAP1, VCAN, VIM, ratios ≧1.5) and 10 down-regulated genes (CDH1, FGFBP1, FOXC2, IL1RN, MMP2, MMP3, NOTCH1, PDGFRB, SOX10, WNT11, ratios ≧−1.5) in the pcDNA3.0-GLIPR-2-transfected cells (Table S1). As shown in Table 2, the CDH1 gene that encodes E-cadherin and N-cadherin is down-regulated (ratio: −1.84), and the VIM gene that encodes the mesenchymal marker vimentin is up-regulated (ratio: 2.08). To further identify the key genes that are involved in an EMT, RT-qPCR evaluation was employed to determine the genes with significant changes, i.e., increases or decreases of at least two-fold (Figure S2). Notably, EGFR gene expression increased in the GLIPR-2-overexpressing HK-2 cells.

**Table 2:** Genes that are up- and down-regulated by the overexpression of GLIPR-2.

Gene Ref Seq.	Gene symbol	Description	Fold change	Function
NM_004360	CDH1	Cadherin 1, type 1, E-cadherin (epithelial)	−1.84	MF: cell adhesion molecule, binding, signal transducer
NM_005228	EGFR	Epidermal growth factor receptor (erythroblastic leukemia viral (v-erb-b) oncogene homolog, avian)	2.52	BP: cell growth and maintenance; MF: signal transducer, binding, catalytic activity
NM_000577	IL1RN	Interleukin 1 receptor antagonist	−3.16	BP: binding, immune and inflammatory responses
NM_002609	PDGFRB	Platelet-derived growth factor receptor,beta polypeptide	−2.21	BP: cell growth and maintenance; MF: signal transducer, binding, catalytic activity
NM_004385	VCAN	Versican	3.16	BP: cell adhesion, migration, and proliferation
NM_003380	VIM	Vimentin	2.08	MF: structural molecule activity, binding

### GLIPR-2 Overexpression Promotes an EMT and Cell Migration through ERK1/2 Activation

**Figure 3:**
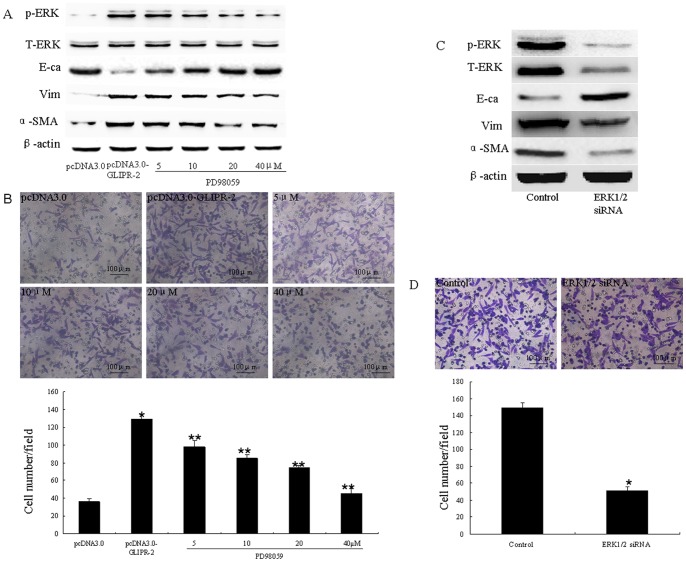
GLIPR-2 overexpression in HK-2 cells promotes an EMT through ERK1/2 activation. (A)Western blot data of EMT markers and ERK1/2 activation in GLIPR-2-overexpressing HK-2 cells. p-ERK1/2 was elevated in the pCDNA3.0-GLIPR-2-transfected HK-2 cells but decreased gradually in a dose-dependent manner with PD98059 treatment; E-cadherin decreased in the pCDNA3.0-GLIPR-2-transfected HK-2 cells but increased gradually in a dose-dependent manner with PD98059 treatment; vimentin and α-smooth muscle actin increased in the pCDNA3.0- GLIPR-2-transfected HK-2 cells but decreased gradually in a dose-dependent manner with PD98059 treatment.(B) Promotion of cell migration by GLIPR-2 overexpression, and the inhibition of cell migration via the abrogation of ERK1/2 activity with PD98059 in the GLIPR-2-transfected group. Two groups of confluent, growth-arrested, and serum-starved HK-2 cells were seeded onto the upper part of the Millicell (8-µm pore size) with various concentrations of PD98059 in the GLIPR-2-transfected group. After incubating the cells for 8 hrs, the number of migrating cells was counted as described above. The data are presented as the mean ± SD. *P<0.01 compared with the pcDNA3.0 group. **P<0.01 compared with the GLIPR-2-transfected group. One of three individual experiments is shown. (C) Western blot data of EMT markers and ERK1/2 activation in the ERK1/2 siRNA-transfected HK-2 cells that overexpressed GLIPR-2. Total-ERK1/2 and p-ERK1/2 decreased and EMT markers were reversed after treatment with the ERK1/2 siRNA. (D) Inhibition of cell migration by ERK1/2 siRNA transfection of the HK-2 cells which overexpressed GLIPR-2. The data are presented as the mean ± SD. *P<0.01 compared with the control groups.

To determine whether the overexpressed GLIPR-2 protein affected the functions of the HK-2 cells, we examined the levels of expression of EMT markers, such as E-cadherin, vimentin and α-SMA in both the GLIPR-2-transfected HK-2 cells and the control cells. As shown in Figure 3 A, the expression of E-cadherin decreased, whereas the levels of vimentin and α-SMA increased in the GLIPR-2-transfected cells. RT-qPCR data illustrating the effect of GLIPR-2 overexpression in HK-2 cells on EMT markers are shown in Figure S3. To confirm the promotion of an EMT, we examined cell migration. As shown in Figure 3 B, after 8 hrs of incubation, the number of migrating cells in the GLIPR-2-transfected group (129.2±7.38/field) was significantly larger than that in the mock-transfected groups (36.0±4.60/field) (n  = 6, *P*<0.01).

Based on the result that GLIPR-2 overexpression promotes EGFR gene expression, we examined whether the mitogen-activated protein kinase (MAPK)/ERK pathway of EGFR-mediated downstream signaling was involved. MAPK cascades, including the MEK-ERK1/2 pathway, are reportedly involved in PTC migration and proliferation. Thus, we examined ERK 1/2 activation by measuring the level of phospho-ERK1/2. As shown in Figure 3 A, the GLIPR-2-transfected HK-2 cells had an increased level of phospho-ERK1/2. The MEK inhibitor PD98059 was applied to abrogate the ERK activation that was induced by the overexpression of GLIPR-2 to determine whether the EMT changes are related to the MEK-ERK1/2 activation. We found that PD98059 reduced the levels of EMT markers and cell migration in a dose-dependent manner (Figure 3 A). To confirm that MAPK/ERK activation is involved in the GLIPR-2-mediated EMT changes, ERK1/2 siRNA and control siRNA were transfected into HK-2 cells that overexpressed GLIPR-2. As shown in Figure 3 C, total- and phospho-ERK1/2 were decreased and the EMT markers were reversed after treatment with ERK1/2 siRNA. In addition, the cell migration was also decreased (Figure 3 D).

### Non-myristoylated GLIPR-2 Overexpression in HK-2 Cells has no Effect on EMT

**Figure 4:**
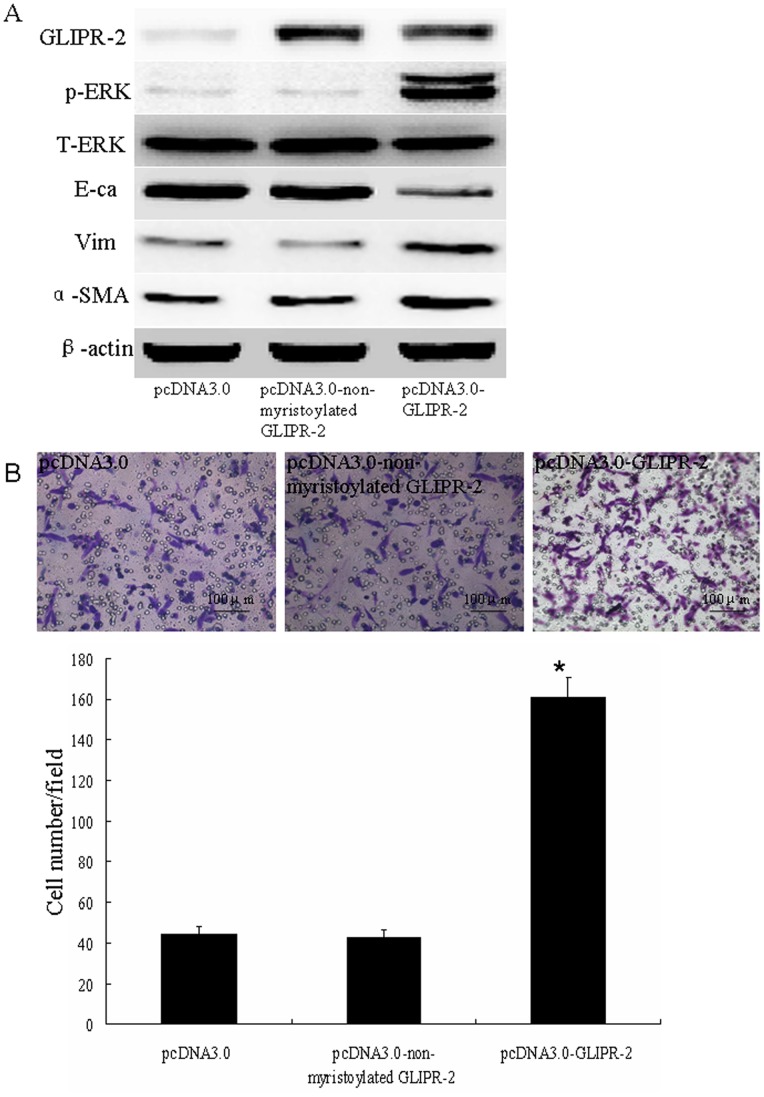
Non-myristoylated GLIPR-2 overexpression has no effect on EMT. (A) Western blot data of EMT markers and ERK1/2 activation in HK-2 cells overexpressing non-myristoylated GLIPR-2. GLIPR-2 expression was elevated in GLIPR-2- and non-myr GLIPR-2-transfected HK-2 cells. However, p-ERK1/2 and EMT markers did not change in the non-myr GLIPR-2-transfected HK-2 cells. The cell migration results were consistent with the Western blot data (B). The data are presented as the mean ± SD. *P<0.01 compared with the pcDNA3.0 group and non-myristoylated GLIPR-2 group.

Because no activity of GLIPR-2 is known, it is difficult to make functionally inactive proteins and subsequently show that the overexpression of the inactive protein does not cause an EMT. It has been revealed that a consensus sequence for myristoylation at the N-terminus of GLIPR-2 was involved in protein-protein interactions, which could provide a mechanism to anchor this protein to the membrane [13], [14]. Thus, non-myristoylated GLIPR-2 was transfected into the HK-2 cells to confirm that this protein has no effect on an EMT. As shown in Figure 4 A, EMT markers and phospho-ERK1/2 levels did not change in the non-myristoylated GLIPR-2 group compared with the mock transfected groups. The cell migration was also unchanged (Figure 4 B).

## Discussion

GLIPR-2 belongs to the PR-1 protein family, which was first identified in plants during the acquisition of resistance to viral infections [16]. The PR-1 proteins contain a sperm-coating protein (SCP) domain (13 kDa) whose function remains unknown [17]. In humans, GLIPR-2 is mainly expressed in the spleen, monocytes and leukocytes, suggesting a role for GLIPR-2 in immunity [13]. Although PR-1 proteins and other mammalian SCP domain-containing proteins are secretory proteins, GLIPR-2 is a non-secretory protein because it lacks a signal peptide and binds negatively charged membranes [18]. Baxter et al. reported that GLIPR-2 was highly expressed in the epithelial cells of the fibrotic kidney accompanied by α-SMA expression in the same areas [11]. Consistent with the results of Baxter et al., we found that GLIPR-2 was abundantly and specifically expressed *in vivo* in the renal tubular epithelial cells of DN kidney tissue samples, which contain the typical progressive renal tubulointerstitial fibrosis characteristic of this disease (Figure 1). Furthermore, we also found that the EMT marker E-cadherin decreased, whereas the vimentin and α-SMA EMT markers were increased in the same regions.

To explore the underlying mechanisms, we established an HK-2 cell line that stably overexpressed GLIPR-2 to determine its effect on the EMT. The data from both animal and *in vitro* studies have indicated that a number of interstitial myofibroblasts are derived from PTCs through an EMT [8], [19]–[21]. PTCs are considered to lose their epithelial features (E-cadherin) and acquire mesenchymal features (α-SMA) during an EMT, which is characterized by increased cell migration and invasion and has recently been identified as type two EMT [9], [22], [23]. The EMT PCR Array analysis indicated that GLIPR-2 overexpression in HK-2 cells increased E-cadherin expression but decreased vimentin expression. Normally, α-SMA expression is low in PTCs, but it is increased when renal fibrosis occurs, which is considered to be evidence for an EMT. GLIPR-2 overexpression also resulted in a significant increase in α-SMA. Additionally, the cell migration assay demonstrated that increased GLIPR-2 expression promoted HK-2 cell migration. Taken together, these results suggest that the high expression of GLIPR-2 in epithelial cells may promote an EMT.

EGFR is the receptor of EGF, which can activate the MEK-ERK-1/2 pathway. Previous studies have shown that phosphorylation of ERK-1/2 by MEK was elevated in PTCs from patients with DN and associated with EMT. Zuo, et al reported that EGFR activation induced a change with an EMT-like phenotype in scc10A cells [24]. Based on the EMT PCR Array analysis, we found that EGFR expression was up-regulated after GLIPR-2 overexpression. Our study indicates that p-ERK-1/2 expression is enhanced in PTCs from patients with DN consistent with the GLIPR-2 expression. To further identify the mechanism underlying the increase in the EMT-like process, we focused on the ERK1/2 pathway, which plays a central role in cell proliferation and differentiation control. We demonstrated that GLIPR-2 overexpression in HK-2 cells promoted activation of the ERK1/2 signaling pathway. Furthermore, we found that PD98059, a specific ERK1/2 inhibitor, inhibited the GLIPR-2-initiated activation of ERK1/2 signaling and cell migration in a dose-dependent manner. To confirm that MAPK/ERK activation was involved in the GLIPR-2-mediated EMT changes, groups of GLIPR-2-transfected cells were transfected with ERK1/2 siRNA. The EMT markers and cell migration were reversed after the ERK1/2 knockdown. Thus, our data strongly confirm the role of ERK1/2 as a downstream regulator in the sequence of events initiated by GLIPR-2, which led to cell migration following an EMT.

In summary, we have shown that GLIPR-2 overexpression in HK-2 cells is likely to contribute significantly to an EMT through ERK1/2 activation followed by enhanced migration. These findings suggest that GLIPR-2 may be involved in PTC EMT to promote cell migration.

## Supporting Information

Figure S1 **RT-PCR analysis of GLIPR-2 mRNA level.** The expression of GLIPR-2 mRNA could not be detected in the parental cells stably transfected with the pcDNA3.0 plasmid, whereas GLIPR-2 expression significantly increased in the cells stably transfected with the pcDNA3.0-GLIPR-2 plasmid.
(TIF)

Figure S2 **Histogram showing the expression values of the selected 6 genes measured by microarray and RT-qPCR.** *P<0.05, calculated by a one-way analysis of variance.
(TIF)

Figure S3 **RT-qPCR data of effect of EMT markers in GLIPR-2-overexpressing HK-2 cells.** E-cadherin decreased in the pCDNA3.0-GLIPR-2-transfected HK-2 cells but increased gradually in a dose-dependent manner with PD98059 treatment; vimentin and α-smooth muscle actin increased in the pCDNA3.0- GLIPR-2-transfected HK-2 cells but decreased gradually in a dose-dependent manner with PD98059 treatment. Data are presented as mean ± SD; *P<0.05, compared with pcDAN3.0 groups; ^#^ *P*<0.01, compared with pCDNA3.0- GLIPR-2 group.
(TIF)

Table S1 **All 84 genes results of RT^2^ Profiler™ PCR Array Human Epithelial to Mesenchymal Transition (PAHS-090A).** CTNNB1, EGFR, ITGAV, SNAI2, SPARC, STEAP1, VCAN and VIM were up-regulated (ratios ≧1.5); CDH1, FGFBP1, FOXC2, IL1RN, MMP2, MMP3, NOTCH1, PDGFRB, SOX10 and WNT11 were down-regulated (ratios ≧−1.5).
(DOC)
