# Supplementary figures and images for: GLIPR-2 Overexpression in HK-2 Cells Promotes Cell EMT and Migration through ERK1/2 Activation
Source: PLoS One. 2013 Mar 13;8(3):e58574. doi: 10.1371/journal.pone.0058574 (PMC3596275; doi:10.1371/journal.pone.0058574)

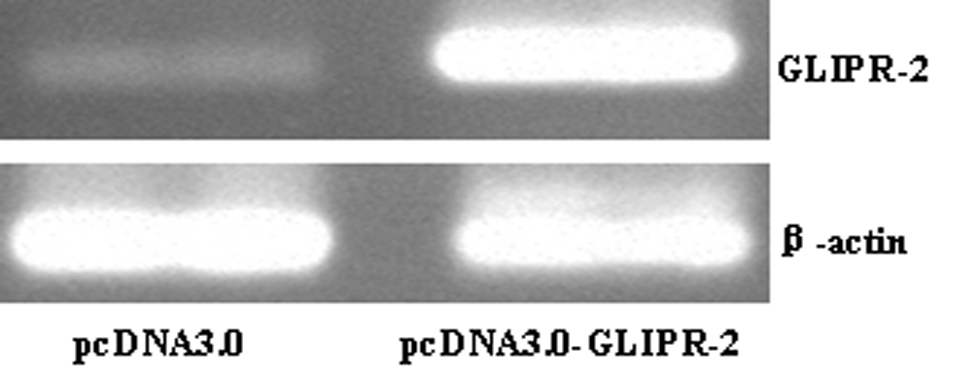

Supplement: Figure S1 — RT-PCR analysis of GLIPR-2 mRNA level. The expression of GLIPR-2 mRNA could not be detected in the parental cells stably transfected with the pcDNA3.0 plasmid, whereas GLIPR-2 expression significantly increased in the cells stably transfected with the pcDNA3.0-GLIPR-2 plasmid. (TIF) [file pone.0058574.s001.tif]

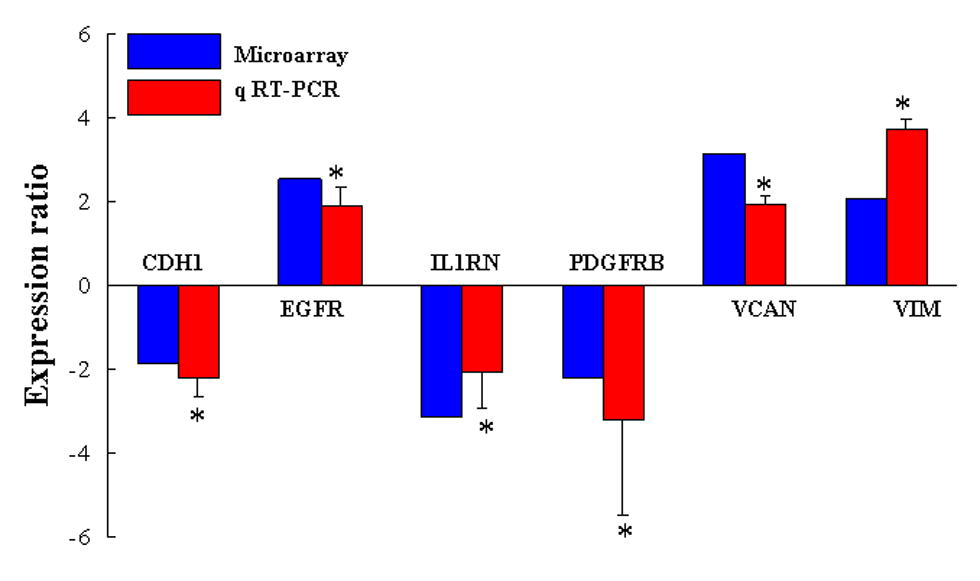

Supplement: Figure S2 — Histogram showing the expression values of the selected 6 genes measured by microarray and RT-qPCR. *P<0.05, calculated by a one-way analysis of variance. (TIF) [file pone.0058574.s002.tif]

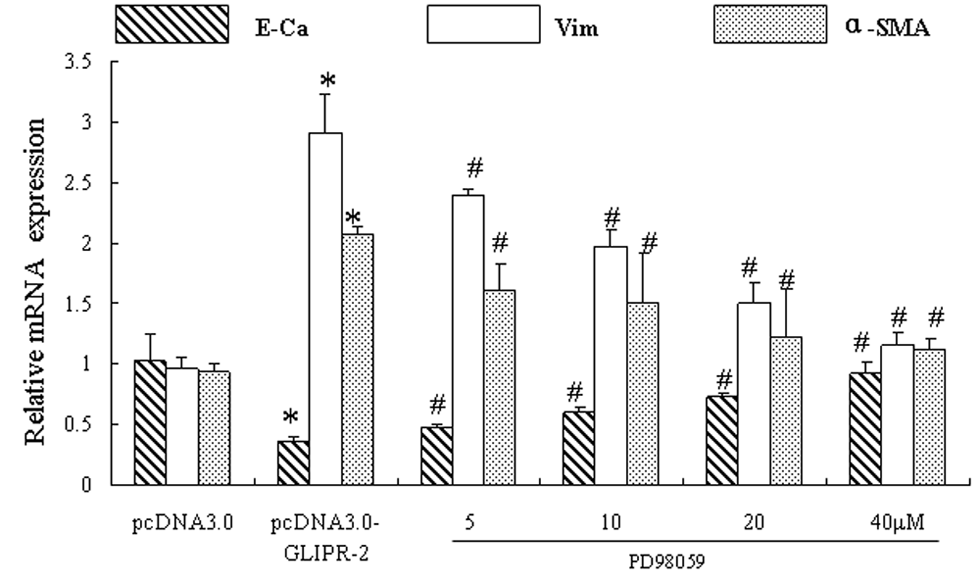

Supplement: Figure S3 — RT-qPCR data of effect of EMT markers in GLIPR-2-overexpressing HK-2 cells. E-cadherin decreased in the pCDNA3.0-GLIPR-2-transfected HK-2 cells but increased gradually in a dose-dependent manner with PD98059 treatment; vimentin and α-smooth muscle actin increased in the pCDNA3.0- GLIPR-2-transfected HK-2 cells but decreased gradually in a dose-dependent manner with PD98059 treatment. Data are presented as mean ± SD; *P<0.05, compared with pcDAN3.0 groups; # P<0.01, compared with pCDNA3.0- GLIPR-2 group. (TIF) [file pone.0058574.s003.tif]
