# Supplementary material for: GLIPR-2 Overexpression in HK-2 Cells Promotes Cell EMT and Migration through ERK1/2 Activation
Source: PLoS One. 2013 Mar 13;8(3):e58574. doi: 10.1371/journal.pone.0058574 (PMC3596275; doi:10.1371/journal.pone.0058574)
Supplement: Table S1 — All 84 genes results of RT2 Profiler™ PCR Array Human Epithelial to Mesenchymal Transition (PAHS-090A). CTNNB1, EGFR, ITGAV, SNAI2, SPARC, STEAP1, VCAN and VIM were up-regulated (ratios ≧1.5); CDH1, FGFBP1, FOXC2, IL1RN, MMP2, MMP3, NOTCH1, PDGFRB, SOX10 and WNT11 were down-regulated (ratios ≧−1.5). (DOC) [file pone.0058574.s004.doc]

**Table S1** All 84 genes results of RT² Profiler™ PCR Array Human Epithelial to Mesenchymal Transition (PAHS-090A).

| Gene Ref Seq. | Gene symbol | Description | Fold change |
| --- | --- | --- | --- |
| NM_024060 | AHNAK | AHNAK nucleoprotein | -1.12 |
| NM_005163 | AKT1 | V-akt murine thymoma viral oncogene homolog 1 | -1.39 |
| NM_006129 | BMP1 | Bone morphogenetic protein 1 | -1.02 |
| NM_001719 | BMP7 | Bone morphogenetic protein 7 | 1.03 |
| NM_004342 | CALD1 | Caldesmon 1 | 1.29 |
| NM_018584 | CAMK2N1 | Calcium/calmodulin-dependent protein kinase II inhibitor 1 | 1.03 |
| NM_001233 | CAV2 | Caveolin 2 | -1.22 |
| NM_004360 | CDH1 | Cadherin 1, type 1, E-cadherin (epithelial) | -1.84 |
| NM_001792 | CDH2 | Cadherin 2, type 1, N-cadherin (neuronal) | -1.07 |
| NM_000089 | COL1A2 | Collagen, type I, alpha 2 | -1.03 |
| NM_000090 | COL3A1 | Collagen, type III, alpha 1 | 1.15 |
| NM_000393 | COL5A2 | Collagen, type V, alpha 2 | -1.12 |
| NM_001904 | CTNNB1 | Catenin (cadherin-associated protein), beta 1, 88kDa | 1.50 |
| NM_004949 | DSC2 | Desmocollin 2 | -1.01 |
| NM_004415 | DSP | Desmoplakin | -1.22 |
| NM_005228 | EGFR | Epidermal growth factor receptor (erythroblastic leukemia viral (v-erb-b) oncogene homolog, avian) | 2.52 |
| NM_001982 | ERBB3 | V-erb-b2 erythroblastic leukemia viral oncogene homolog 3 (avian) | -1.41 |
| NM_000125 | ESR1 | Estrogen receptor 1 | -1.23 |
| NM_144503 | F11R | F11 receptor | -1.26 |
| NM_005130 | FGFBP1 | Fibroblast growth factor binding protein 1 | -1.56 |
| NM_002026 | FN1 | Fibronectin 1 | 1.08 |
| NM_005251 | FOXC2 | Forkhead box C2 (MFH-1, mesenchyme forkhead 1) | -1.57 |
| NM_003507 | FZD7 | Frizzled homolog 7 (Drosophila) | -1.16 |
| NM_004126 | GNG11 | Guanine nucleotide binding protein (G protein), gamma 11 | -1.07 |
| NM_173849 | GSC | Goosecoid homeobox | 1.16 |
| NM_002093 | GSK3B | Glycogen synthase kinase 3 beta | 1.02 |
| NM_001552 | IGFBP4 | Insulin-like growth factor binding protein 4 | -1.47 |
| NM_000577 | IL1RN | Interleukin 1 receptor antagonist | -3.16 |
| NM_004517 | ILK | Integrin-linked kinase | -1.17 |
| NM_002205 | ITGA5 | Integrin, alpha 5 (fibronectin receptor, alpha polypeptide) | 1.16 |
| NM_002210 | ITGAV | Integrin, alpha V (vitronectin receptor, alpha polypeptide, antigen CD51) | 1.68 |
| NM_002211 | ITGB1 | Integrin, beta 1 (fibronectin receptor, beta polypeptide, antigen CD29 includes MDF2, MSK12) | -1.10 |
| NM_000214 | JAG1 | Jagged 1 (Alagille syndrome) | -1.42 |
| NM_000526 | KRT14 | Keratin 14 | 1.39 |
| NM_002276 | KRT19 | Keratin 19 | 1.30 |
| NM_005556 | KRT7 | Keratin 7 | -1.18 |
| NM_005909 | MAP1B | Microtubule-associated protein 1B | 1.12 |
| NM_000248 | MITF | Microphthalmia-associated transcription factor | -1.01 |
| NM_004530 | MMP2 | Matrix metallopeptidase 2 (gelatinase A, 72kDa gelatinase, 72kDa type IV collagenase) | -1.53 |
| NM_002422 | MMP3 | Matrix metallopeptidase 3 (stromelysin 1, progelatinase) | -1.91 |
| NM_004994 | MMP9 | Matrix metallopeptidase 9 (gelatinase B, 92kDa gelatinase, 92kDa type IV collagenase) | 1.27 |
| NM_002444 | MSN | Moesin | -1.09 |
| NM_002447 | MST1R | Macrophage stimulating 1 receptor (c-met-related tyrosine kinase) | -1.34 |
| NM_018055 | NODAL | Nodal homolog (mouse) | -1.19 |
| NM_017617 | NOTCH1 | Notch homolog 1, translocation-associated (Drosophila) | -1.77 |
| NM_015901 | NUDT13 | Nudix (nucleoside diphosphate linked moiety X)-type motif 13 | 1.46 |
| NM_002538 | OCLN | Occludin | -1.05 |
| NM_002609 | PDGFRB | Platelet-derived growth factor receptor, beta polypeptide | -2.21 |
| NM_016445 | PLEK2 | Pleckstrin 2 | -1.34 |
| NM_015704 | PPPDE2 | PPPDE peptidase domain containing 2 | -1.18 |
| NM_005607 | PTK2 | PTK2 protein tyrosine kinase 2 | 1.11 |
| NM_003463 | PTP4A1 | Protein tyrosine phosphatase type IVA, member 1 | 1.29 |
| NM_006908 | RAC1 | Ras-related C3 botulinum toxin substrate 1 (rho family, small GTP binding protein Rac1) | 1.01 |
| NM_002923 | RGS2 | Regulator of G-protein signaling 2, 24kDa | -1.03 |
| NM_000602 | SERPINE1 | Serpin peptidase inhibitor, clade E (nexin, plasminogen activator inhibitor type 1), member 1 | -1.40 |
| NM_003616 | SIP1 | Survival of motor neuron protein interacting protein 1 | -1.12 |
| NM_005901 | SMAD2 | SMAD family member 2 | 1.24 |
| NM_005985 | SNAI1 | Snail homolog 1 (Drosophila) | 1.29 |
| NM_003068 | SNAI2 | Snail homolog 2 (Drosophila) | 1.55 |
| NM_178310 | SNAI3 | Snail homolog 3 (Drosophila) | -1.08 |
| NM_006941 | SOX10 | SRY (sex determining region Y)-box 10 | -1.78 |
| NM_003118 | SPARC | Secreted protein, acidic, cysteine-rich (osteonectin) | 1.50 |
| NM_000582 | SPP1 | Secreted phosphoprotein 1 | 1.07 |
| NM_003150 | STAT3 | Signal transducer and activator of transcription 3 (acute-phase response factor) | 1.44 |
| NM_012449 | STEAP1 | Six transmembrane epithelial antigen of the prostate 1 | 1.74 |
| NM_003200 | TCF3 | Transcription factor 3 (E2A immunoglobulin enhancer binding factors E12/E47) | -1.24 |
| NM_003199 | TCF4 | Transcription factor 4 | -1.05 |
| NM_006528 | TFPI2 | Tissue factor pathway inhibitor 2 | 1.19 |
| NM_000660 | TGFB1 | Transforming growth factor, beta 1 | 1.03 |
| NM_003238 | TGFB2 | Transforming growth factor, beta 2 | -1.44 |
| NM_003239 | TGFB3 | Transforming growth factor, beta 3 | -1.12 |
| NM_003254 | TIMP1 | TIMP metallopeptidase inhibitor 1 | 1.09 |
| NM_003692 | TMEFF1 | Transmembrane protein with EGF-like and two follistatin-like domains 1 | 1.19 |
| NM_178031 | TMEM132A | Transmembrane protein 132A | -1.36 |
| NM_014399 | TSPAN13 | Tetraspanin 13 | -1.05 |
| NM_000474 | TWIST1 | Twist homolog 1 (Drosophila) | -1.07 |
| NM_004385 | VCAN | Versican | 3.16 |
| NM_003380 | VIM | Vimentin | 2.08 |
| NM_033305 | VPS13A | Vacuolar protein sorting 13 homolog A (S. cerevisiae) | -1.30 |
| NM_004626 | WNT11 | Wingless-type MMTV integration site family, member 11 | -1.72 |
| NM_003392 | WNT5A | Wingless-type MMTV integration site family, member 5A | 1.08 |
| NM_032642 | WNT5B | Wingless-type MMTV integration site family, member 5B | -1.02 |
| NM_030751 | ZEB1 | Zinc finger E-box binding homeobox 1 | 1.00 |
| NM_014795 | ZEB2 | Zinc finger E-box binding homeobox 2 | 1.06 |
